# Dietary Polyethylene Glycol Mitigates Tannin-Related Constraints and Enhances Performance in Growing Balkhi Sheep Fed Oak Leaf-Based Diets

**DOI:** 10.3390/vetsci13090947

**Published:** 2026-09-11

**Authors:** Rifat Ullah Jan, Muhammad Farooq, Muhammad Tahir Khan, Mengzhi Wang, Hosameldeen Mohamed Husien, Muhammad Shuaib, Kasim Sakran Abass, Wing-Fu Lai, Ayman A. Swelum

**Affiliations:** 1 College of Animal Sciences and Technology, Yangzhou University, Yangzhou 225009, China; 2 Department of Animal Nutrition, Faculty of Animal Husbandry and Veterinary Science, University of Agriculture, Peshawar 25130, Pakistan; 3 Arid Zone Small Ruminants Research Institute, Ghulam Banda, Kohat 26010, Pakistan; 4 Department of Physiology, Biochemistry, and Pharmacology, College of Veterinary Medicine, University of Kirkuk, Kirkuk 36001, Iraq; 5 School of Food Science and Nutrition, University of Leeds, Leeds LS2 9JT, UK; 6 Department of Animal Production, College of Food and Agriculture Sciences, King Saud University, Riyadh 11451, Saudi Arabia

**Keywords:** oak foliage, PEG-6000, Balkhi sheep, lamb performance, feed conversion, tannin complexation

## Abstract

In hilly regions of Pakistan, particularly South Waziristan, oak (*Quercus baloot*) leaves are a potential locally available feed source, but their tannins may reduce intake and nutrient digestion. Because polyethylene glycol (PEG) binds dietary tannins and can reduce tannin–protein and tannin–enzyme interactions, potentially improving nutrient availability, we evaluated PEG as a practical dietary additive in tannin-rich oak diets. Using practical, non-purified diets, we conducted a 60-day study with thirty-two male Balkhi sheep (3 months old; initial body weight 21.20 ± 0.29 kg). Sheep were stratified by weight and allocated to four diets in a 2 × 2 factorial design: 0% oak + 0% PEG, 45% oak + 0% PEG, 0% oak + 2% PEG, and 45% oak + 2% PEG. We evaluated dry matter and nutrient intake, apparent digestibility, body weight gain, hematology, and blood biochemistry. The 45% oak + 2% PEG diet produced the highest nutrient intake, digestibility, final body weight, and average daily gain, and the lowest Feed conversion ratio. Compared with the 45% oak + 0% PEG diet, average daily gain increased from 77.3 to 181.8 g/day, and dry matter digestibility increased from 41.9% to 57.2%. Endpoint hematology showed no treatment-related abnormalities, while blood urea nitrogen and alanine aminotransferase were significantly lower in sheep receiving 45% oak + 2% PEG than in those receiving the other three diets. Because the diets were practical formulations rather than nutrient-matched mechanistic diets, these findings support the tested oak + PEG diet under local study conditions but do not isolate tannin binding as the sole mechanism.

## Simple Summary

Sheep farmers in the hilly regions of Pakistan, particularly in South Waziristan, frequently experience feed shortages when conventional fodder is unavailable. Oak leaves (*Quercus baloot*) grow abundantly in these areas and could serve as an alternative feed, but their high tannin content often limits intake and nutrient digestion. In this 60-day experiment, thirty-two male Balkhi sheep were assigned to four diets in a 2 × 2 factorial design: a control diet, a diet containing 45% oak leaves, a diet containing 2% polyethylene glycol (PEG), and a diet containing both 45% oak leaves and 2% PEG. The combination of oak leaves and PEG consistently outperformed all other diets, resulting in the highest feed intake, nutrient digestibility, weight gain, and feed efficiency, while the oak-only diet performed the poorest. Blood health indicators remained normal across all treatments, confirming the absence of adverse effects. These findings demonstrate that oak leaves, when supplemented with PEG, represent a viable and effective feed resource for Balkhi sheep during periods of fodder scarcity. All 32 sheep completed the trial with no health issues.

## 1. Introduction

Livestock is a significant part of Pakistan’s agricultural sector and economy, employing many rural people. However, good-quality feed is often limited in the livestock sector, which affects growth, production, and efficiency in ruminants [1,2]. An important browse species available locally in abundance is Oak (*Quercus baloot*), which grows naturally in Khyber Pakhtunkhwa and the South Waziristan region. Oak leaves are fibrous and contain minerals and other nutrients, making them a possible substitute feed ingredient [3,4,5]. However, the feeding value is limited by anti-nutritional factors, especially tannins, which may limit palatability and protein digestibility, as well as inhibit nutrient utilization [6].

The need to explore local feed resources is important because livestock is a major part of Pakistan’s rural economy. According to the Pakistan Economic Survey 2024–25, livestock contributes 63.60% to agricultural value addition and 14.97% to Pakistan’s GDP [7]. At the same time, many livestock farmers continue to face pressure from high feed costs, seasonal fodder shortages, climate shocks, and reduced pasture availability. A recent IPC assessment reported that around 11 million people in Pakistan’s rural areas faced high acute food insecurity between November 2024 and March 2025, and the analysis covered flood-affected and vulnerable rural districts across Balochistan, Sindh, and Khyber Pakhtunkhwa [8]. These conditions underscore the importance of locally available browse resources for small ruminant production. In mountainous areas such as South Waziristan, oak leaves may provide a useful seasonal feed resource when conventional fodder is limited.

Oak leaves are useful in this context because Quercus species grow naturally in mountainous parts of Pakistan, including Khyber Pakhtunkhwa and South Waziristan. However, their use as feed is limited by their tannin content. Oak leaves contain both hydrolyzable and condensed tannins. These compounds can bind dietary proteins and digestive enzymes, reduce palatability, and lower nutrient utilization when present at high levels. In the oak leaves used in this trial, total tannins were 84.8 g/kg DM, with hydrolyzable tannins forming the larger fraction and condensed tannins forming the smaller fraction. This tannin profile provides the biological reason for testing polyethylene glycol (PEG), because PEG can bind tannins and reduce their negative effect on protein digestion and nutrient availability.

Tannins have both beneficial and harmful effects in ruminants, depending on concentration, chemical type, source, and the overall diet [9,10]. A recent meta-analysis of tannin extracts in sheep and cattle identified an optimal inclusion level of approximately 1.5 g/100 g diet DM (15 g/kg DM) for dry matter intake, whereas a threshold of approximately 3 g/100 g DM (30 g/kg DM) was associated with a 5% reduction in intake; the corresponding threshold for weight gain was approximately 2.3 g/100 g DM (23 g/kg DM) [9]. These values, however, were derived from tannin extracts and should not be directly equated with the total tannin concentration of tannin-rich browse. Polyethylene glycol (PEG) is a tannin-binding agent that forms complexes with tannins and can reduce tannin–protein interactions, thereby improving the nutritional utilization of tannin-rich feeds [11,12]. Previous studies have evaluated PEG supplementation with tannin-rich browse in small ruminants, including oak leaves and Acacia-based diets [13,14]. In these studies, Silanikove et al. supplemented goats fed oak leaves with 10 g PEG/day, whereas Brown and Ng’ambi evaluated 0, 23, and 30 g PEG-4000/goat/day in goats receiving Acacia karroo-based diets. In contrast, the present study supplied PEG-6000 (average molecular weight 6000; Merck, Darmstadt, Germany) at 2% of diet dry matter (20 g/kg diet DM). Because the previous studies expressed PEG supplementation as a fixed amount per animal per day, whereas the present study expressed PEG relative to diet DM, these doses are not directly equivalent. However, comparable practical information remains limited for *Quercus baloot*-based diets in Balkhi sheep under the feeding conditions of South Waziristan.

Balkhi sheep are a local breed of meat and wool, and their productivity is limited by poor-quality feed during important growth phases [15]. Thus, enhancing the safe use of local browse species may improve economic and efficient feeding. There is also evidence that tannin-rich feedstuffs can affect animal intake, digestibility, nitrogen metabolism, and performance, but these effects vary depending on tannin type, intake level, and the overall diet [16,17,18].

While earlier studies have reported improved performance in goats and lambs fed tannin-rich browse with PEG supplementation [14,19], practical data remain limited for *Quercus baloot*-based diets in Balkhi sheep under the specific conditions of South Waziristan, where farmers opportunistically use browse during feed shortages.

Therefore, this study evaluated the effects of oak leaves, with and without PEG supplementation, on feed intake, nutrient digestibility, body weight gain, hematology, and blood biochemistry in male Balkhi sheep under local feeding conditions.

## 2. Materials and Methods

### 2.1. Ethical Approval

The protocol for the animal study was approved by the Ethical Committee of the Faculty of Animal Husbandry and Veterinary Sciences, The University of Agriculture, Peshawar, Pakistan. Approval date: 21 March 2024; approval number: 703/PS/UAP. The sheep were observed daily during the experiment, and appropriate veterinary care was provided to reduce stress and maintain animal welfare.

### 2.2. Experimental Design

This 60-day feeding trial evaluates the effects of oak (*Quercus baloot*) leaves and polyethylene glycol (PEG) on intake, digestibility, growth, and blood parameters in male Balkhi sheep.

**Animals and Health Management:** Thirty-two healthy male Balkhi sheep, with an initial body weight of 21.20 ± 0.29 kg (3 months old), were used. Before the start of the experiment, all sheep were subjected to a general clinical health examination by a veterinarian. Health screening included assessment of body condition, appetite, posture, mobility, visible injuries, external parasites, signs of diarrhea, nasal discharge, coughing, lameness, and other clinical signs of disease. Only clinically healthy male Balkhi sheep with no apparent disease, injury, or abnormal behavior were included in the trial.

Before allocation to dietary treatments, all selected sheep were dewormed with ivermectin (Ivovectin^®^, Vetz Pharmaceutical (Pvt.) Ltd., Kotri, Sindh, Pakistan) at a dose of 0.2 mg/kg body weight, administered subcutaneously. The animals were also vaccinated against enterotoxemia using ET-VAC (Sindh Institute of Animal Health, Karachi, Pakistan) and hemorrhagic septicemia (UVAS-HS-VAC, Quality Operations Laboratory, University of Veterinary and Animal Sciences, Lahore, Pakistan) according to the local veterinary vaccination schedule and under the supervision of a veterinarian. After health treatment, sheep were allowed to adapt to the housing and feeding conditions before the start of data collection.

During the experimental period, animals were observed daily for feed intake, behavior, general health condition, and signs of illness or stress. Veterinary care was provided whenever required to maintain animal welfare throughout the study.

**Allocation and Animal Housing:** Sheep were stratified by baseline body weight and then randomly assigned within strata to one of four dietary treatments (*n* = 8 per diet) in a 2 × 2 factorial design to minimize baseline imbalance. The two experimental factors were oak-leaf inclusion (0% or 45% of diet dry matter) and polyethylene glycol (PEG) supplementation (0% or 2% of diet dry matter). The four treatment groups were Diet A (0% oak + 0% PEG; *n* = 8), Diet B (45% oak + 0% PEG; *n* = 8), Diet C (0% oak + 2% PEG; *n* = 8), and Diet D (45% oak + 2% PEG; *n* = 8). For the analysis, the individual sheep was treated as the experimental unit.

Sheep were kept in individual metabolic cages (1.2 m length, 0.8 m width, 0.9 m height) made of galvanized steel, with a slatted plastic floor that allowed for the separate collection of feces and urine. Each open cage had its own feed and water trough. Sheep were maintained under natural photoperiod (approx. 11 h light:13 h dark during Oct–Jan) over the trial. Average ambient temperature and relative humidity were between 8 °C and 22 °C and between 55% and 75%, respectively.

**Feeding and management:** Sheep were fed twice daily (at 08:00 and 16:00 h) and were provided fresh water during this trial. A 10-day adaptation period was implemented before all the measurements were taken. All 32 sheep were used in the final analyses with no animals lost, excluded, or replaced during the 60-day experiment.

**Sample size rationale:** No formal power analysis was done before conducting this experiment. The sample size used (*n* = 8/diet, *n* = 32 total) was designed to provide a factorial feeding trial in individual housing and total fecal collection systems. This number was deemed adequate to detect meaningful differences in treatment in the primary outcomes (feed intake, digestibility, growth) but was not used to estimate small effects or differences between subgroups.

### 2.3. Collection and Processing of Oak Leaves

The oak leaves were collected from trees in the South Waziristan mountains (32.5° N, 69.5° E) during June and July. The mature green leaves were hand-plucked, shade-dried for 10 days with good ventilation, and then chopped into 2–4 cm pieces using a mechanical chopper (9ZR-3.2, Dawn Agro China, Lahore, Pakistan) for easy handling and consumption. A sample of the processed leaves was kept for chemical and tannin analysis. The remaining chopped leaves were stored in clean, dry, woven polypropylene bags at ambient temperature until being used for the diet. Berseem (*Trifolium alexandrinum*) hay, used in the control diet, was collected from the irrigated farms in the same region. Because the oak leaves were collected during June and July, leaves from both collection periods were pooled before diet preparation and chemical analysis. Therefore, the reported tannin profile represents the composite oak-leaf batch used in the feeding trial rather than separate month-specific tannin values. Possible variation in tannin content between collection months is acknowledged as a limitation.

### 2.4. Experimental Diets and Supplementation

The four treatment combinations were arranged in a 2 × 2 factorial design with two levels of oak leaves (0% or 45% of diet dry matter) and two levels of PEG (0% or 2% of diet dry matter). The 45% oak level was selected to represent a nutritionally relevant browse-based diet under local conditions. The 2% PEG level was chosen as a practical tannin-binding additive based on previous studies [19]. PEG-6000 (average molecular weight 6000; Merck, Darmstadt, Germany) was weighed daily for each sheep and mixed thoroughly into the concentrate portion of the relevant diet immediately before the morning feeding to ensure uniform distribution.

The experimental diets were designed based on the nutrient requirements of sheep described by the National Research Council [20]. The main dietary ingredients were Berseem hay, wheat straw, maize grain, mineral mixture, and oak leaves. The diet included 0% or 45% oak leaves (dry matter), and PEG-6000 (average molecular weight 6000; Merck, Darmstadt, Germany) at 0% or 2% of diet dry matter, depending on the experimental treatment combinations. The four diets were designed for practical feeding of growing sheep under local feeding conditions.

#### Analytical Characterization of Oak Leaves and Experimental Diets

Samples of the oak leaves used in the trial and the major feed ingredients were analyzed in triplicate to provide a compositional profile relevant to the present feeding study (Table 1 and Table 2).

Representative composite samples of the offered diets were analyzed in triplicate. Nutrient composition was expressed on a dry matter basis, except for dry matter itself. Non-fibrous carbohydrates (NFC) were calculated by difference as: NFC (g/kg DM) = 1000 − ash − CP − EE − NDF.

### 2.5. Feed Intake and Fecal Collection

Intake measurement: Feed offered and feed refusals were recorded daily for each sheep. Refusals were weighed each morning (07:30–08:00 h) before fresh feed was supplied. Daily dry matter intake (DMI) was calculated as the difference between the dry matter of feed offered and feed refused.

**Digestibility trial:** The total collection of feces over 7 days (from day 54 to day 60 of the experiment) was used to determine apparent digestibility. Sheep were kept in metabolic cages with a partition to separate feces and urine, but the use of feces collection bags ensured complete and quantitative collection of feces and prevented feces from being contaminated or lost during collection. Feces were collected at 08:00 h every morning, and the total fresh fecal output of each sheep was weighed daily. A representative subsample was kept for each animal and stored at −20 °C for further processing. Fecal samples were freeze-dried and subsequently dried at 65 °C to constant weight for determination of dry matter. Feed refusals were collected and weighed daily, and subsampled feed refusals were dried for 48 h at 65 °C and weighed to determine dry matter content.

### 2.6. Laboratory Analysis and Nutrient Digestibility

**Feed and fecal analysis:** Samples of feed, refusals, feces, and oak leaves were analyzed for proximate composition. Dry matter (DM), ash, crude protein (CP), ether extract (EE), and crude fiber (CF) were determined according to the described AOAC procedures. Neutral detergent fiber (NDF) and acid detergent fiber (ADF) were determined in representative feed samples using the Goering and Van Soest method and were used to characterize the fiber composition of the experimental diets.

**Tannin analysis of oak leaves:** Total phenolics and total tannins were measured using the Folin–Ciocalteu method (Folin–Ciocalteu phenol reagent, Merck KGaA, Darmstadt, Germany) described by [21]. Condensed tannins were determined by the n-butanol–(Fisher Chemical, Fisher Scientific, Waltham, MA, USA) and HCl (Merck KGaA, Darmstadt, Germany) method [22]. Hydrolyzable tannins were estimated using the potassium-iodate (Merck KGaA, Darmstadt, Germany) procedure [23]. All tannin analyses were performed in triplicate.

**Digestibility calculation:** Apparent digestibility (%) of the reported nutrient fractions was calculated as:

Apparent digestibility (%) = [(Nutrient intake (g) − Nutrient excreted in feces (g))/Nutrient intake (g)] × 100

### 2.7. Body Weight and Performance Measurements

Body weight was measured at day 0 and at 14-day intervals (days 14, 28, 42, and 60) before morning feeding using a calibrated livestock scale (CS3×6-SB, Mughal Scale, Gujranwala, Pakistan) with a precision of ±0.1 kg. Interim body weights were used to monitor growth progression and animal welfare. The prespecified performance outcomes were final body weight, total live-weight gain, average daily gain calculated from initial and final body weight, and feed conversion ratio. Feed conversion ratio is calculated by dividing the total feed consumed by the total weight gained by the animal. Because repeated body-weight records were not included in the prespecified inferential analysis, slope-based growth modeling was not performed in the present study.

### 2.8. Blood Collection and Analysis

On day 60 of the trial, approximately 10 mL of blood was taken from each sheep under sterile conditions from the jugular vein before the morning feed. Blood was divided into two tubes: one plain serum tube and one EDTA-coated tube (BD Vacutainer, Franklin Lakes, NJ, USA).

**Serum biochemistry:** Serum was separated by centrifugation (Model 2420, Vivid Instrument, Islamabad, Pakistan) at 3000 rpm (1500× *g*) for 15 min at 4 °C within 1 h of collection and stored at −20 °C until analysis. Serum biochemistry variables, including blood urea nitrogen (BUN), glucose, cholesterol, calcium, alanine aminotransferase (ALT), and total protein, were measured using commercial diagnostic kits (HUMAN Gesellschaft für Biochemica und Diagnostica mbH, Wiesbaden, Germany) on a semi-automated chemistry analyzer (Microlab 300, Vital Scientific B.V., Dieren, The Netherlands), following the manufacturers′ instructions.

**Hematology:** Whole blood in EDTA tubes was analyzed within 6 h of collection using an automated veterinary hematology analyzer (Mindray BC-2800Vet, Shenzhen Mindray Bio-Medical Electronics Co., Ltd., Shenzhen, China) for white blood cell count (WBC), red blood cell count (RBC), hemoglobin (Hb), and platelet count.

Blood sampling was performed only at the endpoint; therefore, these variables were interpreted as treatment-group comparisons at day 60 rather than as longitudinal clinical trajectories.

### 2.9. Statistical Analysis

Data were analyzed using two-way analysis of variance (ANOVA) for a 2 × 2 factorial design, with oak-leaf inclusion (0% or 45% of diet DM), PEG supplementation (0% or 2% of diet DM), and their interaction as fixed effects. The statistical model was:

Yijk = μ + Oaki + PEGj + (Oak × PEG) ij + eijk

where Yijk is the dependent variable, μ is the overall mean, Oaki is the main effect of oak, PEGj is the main effect of PEG, (Oak × PEG) ij is the interaction, and eijk is the residual error. The individual sheep was the experimental unit. Stratification by initial body weight was used during allocation to improve baseline balance among treatment groups.

Residuals were checked for approximate normality (Shapiro–Wilk test) and homogeneity of variance (Levene′s test); no serious departures required data transformation. When the interaction or main effects were significant, post hoc multiple comparisons among the four-diet means were performed using Tukey′s honest significant difference (HSD) test. Significance was declared at *p* < 0.05. Data are expressed as mean ± standard error of the mean (SEM).

Productive responses (intake, digestibility, growth) were considered primary outcomes; hematology and serum biochemistry were considered secondary supportive outcomes because they were measured only at the endpoint.

All analyses were performed using IBM SPSS Statistics for Windows, version 26.0 (IBM Corp., Armonk, NY, USA) [24].

## 3. Results

### 3.1. Overview

All 32 sheep completed the 60-day trial without mortality, morbidity, or exclusions. Dietary treatments significantly affected feed intake, digestibility, and growth performance (*p* < 0.05; Table 3, Table 4 and Table 5).

### 3.2. Nutrient Intake

Table 3 presents the primary responses in daily nutrient intake. Diet D (45% oak + 2% PEG) showed the highest dry matter intake, organic matter intake, crude protein intake, and ether extract intake, whereas Diet B (45% oak + 0% PEG) showed the lowest values for these intake variables. Because individual nutrient intakes were calculated from total dry matter intake and diet composition, differences in nutrient intakes should be interpreted mainly as consequences of treatment effects on DMI and the analyzed composition of the diets rather than as independent intake responses. Differences in dietary fiber composition were interpreted using the analyzed NDF and ADF concentrations of the experimental diets.

### 3.3. Nutrient Digestibility

Apparent digestibility coefficients are shown in Table 4. Diet D had the highest dry matter digestibility, organic matter digestibility, crude protein digestibility, and ether extract digestibility, whereas Diet B showed the lowest values. The oak × PEG interaction was significant for these digestibility variables, indicating that PEG’s effect depended on the presence of oak leaves in the diet. Ash digestibility was not reported because ash represents a mixed mineral residue rather than a single digestible nutrient fraction.

### 3.4. Growth Performance

Growth performance outcomes are summarized in Table 5. Diet D produced the highest final body weight (32.10 kg) and average daily gain (181.8 g/day), whereas Diet B produced the lowest final body weight and average daily gain. A lower feed conversion ratio indicates better feed efficiency. Diet B had the highest feed conversion ratio (19.53), indicating poorer feed efficiency, while Diet D had the numerically lowest value (8.85), although it was in the same Tukey group as Diets A and C.

### 3.5. Hematology and Blood Biochemistry

#### 3.5.1. Blood Hematology

Table 6 presents endpoint hematological variables. No significant differences among diets were observed for white blood cell count (WBC; range 6.47–6.63 × 10^3^/µL), red blood cell count (RBC; 9.55–9.71 × 10^6^/µL), or hemoglobin (Hb; 12.36–12.94 g/dL). Platelet count showed a significant main effect of PEG (*p* = 0.015), with numerically higher values in PEG-supplemented diets (Diet C: 918 × 10^3^/µL; Diet D: 951 × 10^3^/µL) compared with non-PEG diets (Diet A: 909 × 10^3^/µL; Diet B: 896 × 10^3^/µL). There was no significant oak main effect or oak × PEG interaction for any hematological variable.

All values fell within reference ranges reported for healthy growing lambs (WBC: 4–12 × 10^3^/µL; RBC: 8–12 × 10^6^/µL; Hb: 9–15 g/dL; platelets: 200–1000 × 10^3^/µL).

#### 3.5.2. Blood Biochemistry

Endpoint serum biochemistry results are presented in Table 7. Blood urea nitrogen (BUN) and alanine aminotransferase (ALT) were significantly lower (*p* < 0.05) in Diet D (BUN: 3.18 mmol/L; ALT: 28.2 IU/L) compared with the other three diets (BUN range: 3.77–3.94 mmol/L; ALT range: 30.4–31.2 IU/L). Factorial analysis showed significant oak, PEG, and oak × PEG effects for BUN (*p* = 0.009, *p* < 0.001, *p* < 0.001, respectively), and significant PEG and oak × PEG effects for ALT (*p* = 0.007, *p* = 0.011). Total protein was numerically highest in Diet D (6.58 g/dL), but neither the PEG main effect (*p* = 0.055) nor the interaction (*p* = 0.098) reached significance. Calcium, glucose, and cholesterol did not differ among diets (*p* > 0.05 for all factorial terms).

## 4. Discussion

The objective of this study was to evaluate whether locally available oak-based rations, with or without PEG supplementation, could support intake, digestibility, growth, and blood parameters in male Balkhi sheep under practical feeding conditions in South Waziristan. The main finding was that Diet D (45% oak leaves + 2% PEG) produced the highest dry matter intake, nutrient digestibility, average daily gain, and final body weight, whereas Diet B (45% oak leaves + 0% PEG) generally produced the lowest values for these outcomes. The significant oak × PEG interactions observed for intake, digestibility, and growth outcomes indicate that the effect of oak inclusion depended on PEG supplementation. However, because the diets were practical formulations rather than nutrient-matched mechanistic controls, the superiority of Diet D should be interpreted as the response to this tested whole-diet formulation rather than as proof that tannin binding was the only mechanism involved.

### 4.1. Digestibility and Intake Responses

This increase in the digestibility of dry matter and crude protein in Diet D agrees with previous reports [25,26,27], which found a greater digestibility of tannic-bearing browse for small ruminants when PEG was added to the ration. Although Diet D had the highest dry matter digestibility (DMD) among the treatments, the absolute DMD value remained moderate. This may be explained by the high structural fiber content of oak leaves, particularly their NDF and ADF fractions, and by possible residual tannin activity despite PEG supplementation. PEG can reduce tannin-related constraints, but it may not fully remove the physical limitation imposed by fibrous cell-wall material or completely neutralize tannin interactions in a complex practical diet. Therefore, the oak + PEG diet should be interpreted as an improved browse-based ration rather than as a highly digestible conventional finishing diet. The biological action of PEG is related to its ability to form complexes with tannins, thereby reducing tannin binding with dietary proteins and digestive enzymes. This interpretation is consistent with earlier work showing that tannin-binding agents such as PEG and polyvinylpyrrolidone can modify fermentation responses of tannin-rich feed materials in vitro [28]. This mechanism is reinforced by the many significant interactions between the two factors (oak and PEG) for the digestibility variables (Table 4). Direct measurement of tannin activity, rumen fermentation, and microbial populations was not conducted in the present experiment; however, the mechanism is speculative. In a recent study of goats fed neem leaves (with PEG), the combined treatment resulted in the highest intake, digestibility, and average daily gain, and the study also measured the rumen fermentation and microbial responses [27]. In a broader context, recent meta-analyses indicate that productive responses to tannic diets vary with tannin source, dose, and type of overall diet and that this is part of the reason for the differences in productive responses that occur in small ruminants when fed on a browse-based diet [17].

Daily nutrient intakes were calculated from individual dry matter intake and the analyzed nutrient composition of the respective experimental diet. The main intake finding was that the oak + PEG diet improved total dry matter intake and crude protein intake compared with the oak-only diet, suggesting that PEG improved the acceptability and utilization of the oak-containing ration.

### 4.2. Growth Performance

In this experiment, 45% oak leaves with 2% PEG was the best diet for growth. Diet D had the lowest Feed conversion ratio (8.85) and the highest average daily gain (181.8 g/day), while Diet B had the poorest feed efficiency (19.53) and the lowest gain (77.3 g/day). The improvement in ADG from Diet B to Diet D (77.3 to 181.8 g/day) is not only statistically significant but also practically meaningful for smallholder farmers, as it represents an approximately doubled daily weight gain over the 60 days. The PEG main effect was significant for all growth variables, while the oak main effect was not (Table 5), further supporting that the benefit of oak leaves depends entirely on concurrent PEG supplementation within the tested formulations. Although Diet D produced the best growth response among the tested diets, the Feed conversion ratio observed in this study was still relatively high. This indicates that oak-leaf-based diets should not be presented as high-efficiency finishing rations for young sheep. Their main value lies in their availability as a locally sourced feed option during periods of fodder scarcity. The oak-only diet produced the poorest feed efficiency, showing that high oak inclusion without tannin mitigation may limit productivity. PEG improved feed conversion in the oak-based diet, but its practical value depends on cost, availability, and comparison with alternative feeds.

### 4.3. Blood Biochemistry and Health Indicators

Endpoint serum biochemistry did not show any negative pattern in Diet D. Blood urea nitrogen (BUN) was significantly lower in Diet D (3.18 mmol/L) compared with the other diets (3.77–3.94 mmol/L). Lower BUN typically indicates improved nitrogen utilization or reduced protein catabolism, and has been reported in some tannin-containing diets for small ruminants [15,21]. The lower BUN concentration may also suggest reduced ruminal deamination of dietary amino acids. Tannins can bind dietary proteins and reduce ruminal protein degradation, while PEG may reduce excessive tannin-protein binding and improve the availability of usable protein. Therefore, lower BUN in the oak + PEG group may reflect more efficient nitrogen use and possibly greater bypass protein supply. However, rumen ammonia, microbial protein synthesis, and post-ruminal protein flow were not measured, so this interpretation should remain cautious. This lower BUN is consistent with Diet D, which has higher crude protein digestibility than Diet B (59.0% vs. 42.5%, respectively; Table 4). Alanine aminotransferase (ALT) also showed lower values in Diet D (28.2 IU/L) than in other diets (30.4–31.2 IU/L). ALT is a hepatocellular integrity marker that was within the normal range in sheep (typical reference range 20–40 IU/L); the small difference in numbers is unlikely to be clinically important. However, total protein was numerically highest in Diet D (6.58 g/dL); this was not statistically significant (PEG: *p* = 0.055; interaction: *p* = 0.098).

No significant differences were observed for WBC, RBC, or Hb among the dietary treatments (*p* > 0.05). Platelet count showed a significant main effect of PEG supplementation (*p* = 0.015), with numerically higher platelet counts in the PEG-supplemented diets (Diet C and Diet D) than in the non-PEG diets (Diet A and Diet B). However, all hematological values remained within the reference ranges reported for healthy growing lambs. Thus, the observed platelet difference was not accompanied by evidence of a clinically abnormal hematological profile.

These data should be interpreted only as differences between treatment groups at day 60, not as physiological trajectories over time, since samples were collected only at this endpoint. Blood samples were not collected at baseline; therefore, pre-existing differences cannot be excluded, but stratified randomization indicates an unlikely large pre-existing difference.

### 4.4. Practical Implications, Limitations, and Future Research

In areas with limited feed resources, such as South Waziristan, farmers may use browse as an alternative feed source during periods when conventional fodder is scarce. The present study suggests that oak leaves can be used more effectively in male Balkhi sheep when their tannin-related constraints are mitigated with PEG. Under the tested conditions, the formulation containing 45% oak leaves and 2% PEG produced favorable intake, digestibility, growth, and feed-efficiency responses. However, wider adoption should be considered carefully because the cost, availability, and transportation of PEG may limit its practical use on smallholder farms. Therefore, its use should be evaluated according to local feed availability and the additional production benefits obtained relative to the cost of supplementation.

Several limitations should be considered when interpreting these findings. The study evaluated only one level of oak leaves and one level of PEG, used a relatively small number of animals per treatment (*n* = 8), and was conducted over a 60-day feeding period. In addition, the diets were practical treatment formulations rather than strictly nutrient-matched mechanistic controls; therefore, some responses may reflect differences in overall diet composition in addition to PEG-mediated mitigation of tannin constraints. NDF intake and digestibility were not measured, which could have provided further insight into the effects of dietary fiber differences among the experimental diets. Blood variables were measured only at the endpoint, and rumen fermentation, microbial responses, and direct measures of tannin activity were not assessed. Furthermore, all animals included in the study were male, and the findings should therefore not be generalized to female sheep or breeding flocks without further validation.

Future studies should evaluate multiple oak and PEG inclusion levels and use nutrient-matched comparison diets to better distinguish tannin-related effects from differences in overall diet composition. Longer and larger studies incorporating rumen fermentation measurements, repeated blood sampling, economic evaluation, and female sheep would further clarify the biological and practical value of oak + PEG diets under field conditions.

## 5. Conclusions

This study suggests that oak leaves can be used more effectively in male Balkhi sheep diets when supplemented with PEG. Under the tested conditions, the diet containing 45% oak leaves and 2% PEG produced the most favorable intake, digestibility, growth, and feed efficiency among the four diets, whereas the oak-only diet showed the poorest response. These findings indicate that PEG can reduce the practical feeding constraints associated with tannin-rich oak leaves, although the response should be interpreted as a whole-diet effect rather than proof of tannin binding as the only mechanism.

Practically, PEG should be mixed thoroughly with the concentrate portion immediately before feeding, especially when oak leaves form a large part of the ration. Based on the tested formulation, 45% oak leaves with 2% PEG improved performance under controlled local conditions; however, this should not be generalized to other oak or PEG levels without dose–response testing.

## Figures and Tables

**Table 1:** Compositional and phenolic profile of oak leaves used in the feeding trial (mean ± SD).

Variable	Value	Unit
Dry matter	943.8 ± 2.4	g/kg as-fed
Crude protein	88.6 ± 4.1	g/kg DM
Neutral detergent fiber	526.0 ± 6.2	g/kg DM
Acid detergent fiber	315.8 ± 5.1	g/kg DM
Ash	58.7 ± 2.6	g/kg DM
Total phenolics	96.4 ± 4.0	g/kg DM
Total tannins	84.8 ± 3.5	g/kg DM
Condensed tannins	13.6 ± 0.5	g/kg DM
Hydrolysable tannins	71.2 ± 3.0	g/kg DM

Note: DM = dry matter. Values are the mean ± SD of triplicate laboratory analyses of the composite oak-leaf sample used in the feeding trial; SD therefore reflects analytical variation, not animal-level biological variation.

**Table 2:** Representative nutrient composition of the offered experimental diets (mean ± SD, g/kg DM unless otherwise stated).

Variable	Diet A	Diet B	Diet C	Diet D	Unit
Dry matter	914.2 ± 3.1	920.5 ± 2.8	915.0 ± 3.0	921.3 ± 2.6	g/kg as-fed
Organic matter	914.8 ± 1.6	905.0 ± 1.8	916.2 ± 1.7	914.8 ± 1.7	g/kg DM
Crude protein	134.8 ± 2.1	130.1 ± 2.4	135.7 ± 2.2	145.2 ± 2.6	g/kg DM
Ether extract	55.2 ± 1.1	49.7 ± 1.0	56.1 ± 1.1	54.9 ± 1.1	g/kg DM
Ash	85.2 ± 1.6	95.0 ± 1.8	83.8 ± 1.5	85.2 ± 1.6	g/kg DM
Non-fibrous carbohydrates (NFC)	262.8 ± 6.0	212.8 ± 6.9	264.3 ± 5.9	206.1 ± 6.6	g/kg DM
Neutral detergent fiber	462.0 ± 5.3	512.4 ± 6.1	460.1 ± 5.1	508.6 ± 5.8	g/kg DM
Acid detergent fiber	286.4 ± 4.2	332.1 ± 4.8	285.8 ± 4.1	329.8 ± 4.6	g/kg DM

Note: Values are mean ± SD of triplicate analyses of representative composite diet samples. SD reflects analytical variation of the composite samples rather than biological variation among animals. DM = dry matter. Diet A = 0% oak + 0% PEG; Diet B = 45% oak + 0% PEG; Diet C = 0% oak + 2% PEG; Diet D = 45% oak + 2% PEG. NFC = non-fibrous carbohydrates; NFC = 1000 − ash − CP − EE − NDF.

**Table 3:** Intake and factorial *p*-values of Balkhi sheep fed diets containing oak (*Quercus baloot*) leaves and PEG.

Nutrient Intake (g/Day)	Actual Data/Treatment Means				Error Estimate	Significance/Factorial *p*-Values		
Response Variable	Diet A	Diet B	Diet C	Diet D	SEM	Oak	PEG	Oak × PEG
Dry matter intake (g/day)	1432.00 ^b^	1368.00 ^c^	1444.00 ^b^	1584.00 ^a^	12.9	0.006	<0.001	<0.001
Organic matter intake (g/day)	1310.00 ^b^	1238.00 ^c^	1323.00 ^b^	1449.00 ^a^	9.6	0.009	<0.001	<0.001
Crude protein intake (g/day)	193.00 ^b^	178.00 ^c^	196.00 ^b^	230.00 ^a^	3.4	0.009	<0.001	<0.001
Ether extract intake (g/day)	79.00 ^b^	68.00 ^c^	81.00 ^b^	87.00 ^a^	1.6	0.129	<0.001	<0.001

Note: Means within a row with different superscript letters differ significantly according to Tukey’s test (*p* < 0.05). SEM = pooled standard error of the mean. Oak = main effect of oak-leaf inclusion; PEG = main effect of polyethylene glycol supplementation. Diet A (0% oak + 0% PEG; *n* = 8), Diet B (45% oak + 0% PEG; *n* = 8), Diet C (0% oak + 2% PEG; *n* = 8), and Diet D (45% oak + 2% PEG; *n* = 8).

**Table 4:** Apparent digestibility and factorial *p*-values for Balkhi sheep fed oak (*Quercus baloot*) leaves and polyethylene glycol (PEG).

	Actual Data/Treatment Means				Error Estimate	Significance/Factorial *p*-Values		
Digestibility (%)	Diet A	Diet B	Diet C	Diet D	SEM	Oak	PEG	Oak × PEG
Dry matter digestibility (%)	45.60 ^b^	41.90 ^c^	46.10 ^b^	57.20 ^a^	0.82	<0.001	<0.001	<0.001
Organic matter digestibility (%)	62.70 ^b^	58.60 ^c^	63.00 ^b^	72.10 ^a^	0.91	0.010	<0.001	<0.001
Crude protein digestibility (%)	46.40 ^b^	42.50 ^c^	47.00 ^b^	59.00 ^a^	0.88	<0.001	<0.001	<0.001
Ether extract digestibility (%)	55.30 ^b^	51.90 ^c^	55.90 ^b^	68.00 ^a^	0.90	<0.001	<0.001	<0.001

Note: Means within a row with different superscript letters differ significantly according to Tukey’s test (*p* < 0.05). SEM = pooled standard error of the mean. Diet A (0% oak + 0% PEG; *n* = 8), Diet B (45% oak + 0% PEG; *n* = 8), Diet C (0% oak + 2% PEG; *n* = 8), and Diet D (45% oak + 2% PEG; *n* = 8).

**Table 5:** Feed efficiency and growth performance of Balkhi sheep fed oak leaves and PEG.

	Actual Data/Treatment Means				Error Estimate	Significance/Factorial *p*-Values		
Parameter	Diet A	Diet B	Diet C	Diet D	SEM	Oak	PEG	Oak × PEG
Final body weight (kg)	27.92 ^b^	25.82 ^c^	28.34 ^b^	32.10 ^a^	0.47	0.088	<0.001	<0.001
Average daily gain (g/day)	112.0 ^b^	77.3 ^c^	118.3 ^b^	181.8 ^a^	8.1	0.085	<0.001	<0.001
Feed conversion ratio	13.35 ^b^	19.53 ^a^	12.68 ^b^	8.85 ^b^	1.55	0.456	0.001	0.003

Note: Means within a row with different superscript letters differ significantly according to Tukey’s test (*p* < 0.05). SEM = pooled standard error of the mean. Diet A (0% oak + 0% PEG; *n* = 8), Diet B (45% oak + 0% PEG; *n* = 8), Diet C (0% oak + 2% PEG; *n* = 8), and Diet D (45% oak + 2% PEG; *n* = 8).

**Table 6:** Blood hematology means and factorial *p*-values for Balkhi sheep fed oak (*Quercus baloot*) leaves and PEG.

	Actual Data/Treatment Means				Error Estimate	Significance/Factorial *p*-Values		
Blood Hematology	Diet A	Diet B	Diet C	Diet D	SEM	Oak	PEG	Oak × PEG
WBC (10^3^/µL)	6.52	6.47	6.55	6.63	0.11	0.893	0.395	0.559
RBC (10^6^/µL)	9.61	9.55	9.64	9.71	0.13	0.970	0.471	0.621
Hb (g/dL)	12.50	12.36	12.58	12.94	0.17	0.523	0.062	0.153
Platelets (10^3^/µL)	909.0	896.0	918.0	951.0	12.4	0.427	0.015	0.074

Note: Diet A–D columns present endpoint treatment means, SEM presents the pooled standard error of the mean, and Oak, PEG, and Oak × PEG columns present factorial *p*-values from the two-way ANOVA. WBC = white blood cells; RBC = red blood cells; Hb = hemoglobin. Values are endpoint treatment means. SEM = pooled standard error of the mean. Oak = main effect of oak-leaf inclusion; PEG = main effect of polyethylene glycol supplementation; Oak × PEG = interaction. Diet A (0% oak + 0% PEG; *n* = 8), Diet B (45% oak + 0% PEG; *n* = 8), Diet C (0% oak + 2% PEG; *n* = 8), and Diet D (45% oak + 2% PEG; *n* = 8).

**Table 7:** Blood biochemistry and factorial *p*-values for Balkhi sheep fed oak (*Quercus baloot*) leaves and polyethylene glycol (PEG).

	Actual Data/Treatment Means				Error Estimate	Significance/Factorial *p*-Values		
Blood Biochemistry	Diet A	Diet B	Diet C	Diet D	SEM	Oak	PEG	Oak × PEG
Total protein (g/dL)	6.44	6.32	6.46	6.58	0.07	1.000	0.055	0.098
Calcium (mmol/L)	2.43	2.49	2.44	2.58	0.05	0.055	0.326	0.430
BUN (mmol/L)	3.80 ^a^	3.94 ^a^	3.77 ^a^	3.18 ^b^	0.08	0.009	<0.001	<0.001
ALT (IU/L)	30.5 ^a^	31.2 ^a^	30.4 ^a^	28.2 ^b^	0.53	0.168	0.007	0.011
Glucose (mmol/L)	2.44	2.40	2.49	2.61	0.09	0.660	0.160	0.382
Cholesterol (mmol/L)	1.13	1.10	1.14	1.21	0.04	0.621	0.145	0.222

Means within a row with different superscript letters are significantly different according to Tukey’s test, *p* < 0.05. SEM, pooled standard error of the mean; Oak, main effect of oak-leaf inclusion, 0% vs. 45% of diet dry matter; PEG, main effect of polyethylene glycol supplementation, 0% vs. 2% of diet dry matter; Oak × PEG, interaction. BUN, blood urea nitrogen; ALT, alanine aminotransferase. Diet A (0% oak + 0% PEG; *n* = 8), Diet B (45% oak + 0% PEG; *n* = 8), Diet C (0% oak + 2% PEG; *n* = 8), and Diet D (45% oak + 2% PEG; *n* = 8).

## Data Availability

The original contributions presented in this study are included in the article. Further inquiries can be directed to the corresponding author(s).

## References

[1] Alimi N., Assani A.S., Sanni Worogo H., Baco N.M., Traoré I.A. (2024). Livestock feed resources used as alternatives during feed shortages and their impact on the environment and ruminant performance in West Africa: A systematic review. Front. Vet. Sci..

[2] Boudalia S., Smeti S., Dawit M., Senbeta E.K., Gueroui Y., Dotas V., Bousbia A., Symeon G.K. (2024). Alternative approaches to feeding small ruminants and their potential benefits. Animals.

[3] Muhammad N., Castillejo M.Á., Rey M.-D., Jorrín-Novo J.V. (2023). An overview of oak species in Pakistan: Past, present, and future research perspectives. Forests.

[4] Rahman A., Khan N., Bräuning A., Ullah R., Rahman I.U. (2022). Effects of environmental and spatial gradients on *Quercus*-dominated Mountain forest communities in the Hindu-Kush ranges of Pakistan. Saudi J. Biol. Sci..

[5] Taib M., Rezzak Y., Bouyazza L., Lyoussi B. (2020). Medicinal uses, phytochemistry, and pharmacological activities of *Quercus* species. Evid.-Based Complement. Altern. Med..

[6] Ullah H., Badshah L. (2024). The ethnobotanical heritage of Lotkuh, a high-altitude tribal haven of Chitral, the Eastern Hindu Kush, Pakistan. J. Ethnobiol. Ethnomed..

[7] Finance Division, Government of Pakistan (2025). Pakistan Economic Survey 2024–25: Highlights.

[8] Integrated Food Security Phase Classification (2025). Pakistan: Acute Food Insecurity Analysis, November 2024–July 2025.

[9] Ahmed O., Hassen A., Lehloenya K. (2025). Tannin extract dietary thresholds for preventing unacceptable suppression in intake, digestibility, and growth in sheep and cattle: A meta-analysis. S. Afr. J. Anim. Sci..

[10] Besharati M., Maggiolino A., Palangi V., Kaya A., Jabbar M., Eseceli H., De Palo P., Lorenzo J.M. (2022). Tannin in ruminant nutrition. Molecules.

[11] Makkar H., Blümmel M., Becker K. (1995). Formation of complexes between polyvinyl pyrrolidones or polyethylene glycols and tannins, and their implication in gas production and true digestibility in in vitro techniques. Br. J. Nutr..

[12] Getachew G., Makkar H., Becker K. (2000). Effect of polyethylene glycol on in vitro degradability ofnitrogen and microbial protein synthesis fromtannin-rich browse and herbaceous legumes. Br. J. Nutr..

[13] Silanikove N., Gilboa N., Nitsan Z. (1997). Interactions among tannins, supplementation and polyethylene glycol in goats given oak leaves: Effects on digestion and food intake. Anim. Sci..

[14] Brown D., Ng’ambi J.W. (2017). Effect of polyethylene glycol 4000 supplementation on the performance of yearling male Pedi goats fed dietary mixture levels of *Acacia karroo* leaf meal and *Setaria verticillata* grass hay. Trop. Anim. Health Prod..

[15] Naqvi A., Mahmood S., Vahidi S., Abbas S., Utsunomiya Y., Garcia J., Periasamy K. (2017). Assessment of genetic diversity and structure of major sheep breeds from Pakistan. Small Rumin. Res..

[16] Souza V., Almeida K., Rosenstein P., Desrues O., Panciroli N., Kebreab E. (2025). Impact of tannin-based additives on animal performance and enteric methane emissions in dairy and beef cattle: A meta-analysis. J. Dairy Sci..

[17] Yanza Y.R., Fitri A., Suwignyo B., Hidayatik N., Kumalasari N.R., Irawan A., Jayanegara A. (2021). The utilisation of tannin extract as a dietary additive in ruminant nutrition: A meta-analysis. Animals.

[18] Zhu X., Dou X., Su T., Ye L., Zhang L., Liu H., Han D. (2025). Microbiomic and metabolomic insights into the roles of hydrolysable versus condensed tannins on the growth performance, nutrient digestion, and rumen fermentation in liaoning cashmere goats. Microorganisms.

[19] Chaurasiya A. (2018). Effect of feeding oak (*Quercus leucotrichophora*) leaves on haematological and biochemical parameters of parasitic infected goat in Kumaon hills. Int. J. Agric. Sci..

[20] National Research Council (2001). Nutrient Requirements of Small Ruminants: Sheep, Goats, Cervids, and New World Camelids.

[21] Makkar H.P. (2013). Quantification of Tannins in Tree and Shrub Foliage: A Laboratory Manual.

[22] Porter L.J., Hrstich L.N., Chan B.G. (1985). The conversion of procyanidins and prodelphinidins to cyanidin and delphinidin. Phytochemistry.

[23] Hartzfeld P.W., Forkner R., Hunter M.D., Hagerman A.E. (2002). Determination of hydrolyzable tannins (gallotannins and ellagitannins) after reaction with potassium iodate. J. Agric. Food Chem..

[24] IBM Corp (2019). IBM SPSS Statistics for Windows.

[25] Adejoro F.A., Hassen A., Akanmu A.M. (2019). Effect of lipid-encapsulated acacia tannin extract on feed intake, nutrient digestibility and methane emission in sheep. Animals.

[26] Silanikove N., Gilboa N., Nir I., Perevolotsky A., Nitsan Z. (1996). Effect of a daily supplementation of polyethylene glycol on intake and digestion of tannin-containing leaves (*Quercus calliprinos*, *Pistacia lentiscus*, and *Ceratonia siliqua*) by goats. J. Agric. Food Chem..

[27] Taethaisong N., Paengkoum S., Kaewwongsa W., Onjai-Uea N., Thongpea S., Paengkoum P. (2023). The effect of neem leaf supplementation on growth performance, rumen fermentation, and ruminal microbial population in goats. Animals.

[28] Besharati M., Taghizadeh A. (2011). Effect of Tannin-Binding Agents (Polyethylene Glycol and Polyvinylpyrrolidone) Supplementation on In Vitro Gas Production Kinetics of Some Grape Yield Byproducts. Int. Sch. Res. Not..

